# Optimization of Gluten-Free Bread Formulation Using Whole Sorghum-Based Flour by Response Surface Methodology

**DOI:** 10.3390/foods14173113

**Published:** 2025-09-05

**Authors:** Melissa Rodríguez-España, Claudia Yuritzi Figueroa-Hernández, Mirna Leonor Suárez-Quiroz, Fátima Canelo-Álvarez, Juan de Dios Figueroa-Cárdenas, Oscar González-Ríos, Patricia Rayas-Duarte, Zorba Josué Hernández-Estrada

**Affiliations:** 1 Tecnológico Nacional de México/Instituto Tecnológico de Veracruz, Miguel Ángel de Quevedo 2779, Col. Formando Hogar, Veracruz C.P. 91897, Mexico; d21020001@veracruz.tecnm.mx (M.R.-E.); claudia.fh@veracruz.tecnm.mx (C.Y.F.-H.); mirna.sq@veracruz.tecnm.mx (M.L.S.-Q.); oscar.gr@veracruz.tecnm.mx (O.G.-R.); 2 Centro de Investigación y de Estudios Avanzados del IPN (CINVESTAV Unidad Querétaro), Libramiento Norponiente 2000, Fracc. Real de Juriquilla, Querétaro C.P. 76230, Mexico; fatima.canelo@cinvestav.mx (F.C.-Á.); jfigueroa@cinvestav.mx (J.d.D.F.-C.); 3 Department of Biochemistry and Molecular Biology, Robert M. Kerr Food and Agricultural Products Center, Oklahoma State University, Stillwater, OK 74078, USA

**Keywords:** sorghum, optimization formulations, response surface methodology, gluten-free bread, bread quality

## Abstract

The growing awareness of celiac disease and gluten sensitivities has generated interest in gluten-free products. Whole sorghum (*Sorghum bicolor*) is an excellent source of nutrients and is gluten-free. However, the absence of gluten makes it technologically challenging to produce leavened products. This research aims to utilize a response surface methodology to optimize the specific loaf volume and crumb firmness of a whole sorghum-based gluten-free bread formulation, evaluating different levels of milk powder, egg white, yeast, sugar, psyllium husk powder, xanthan gum, and soy lecithin. The models fit achieved an $R^{2}\geq 80\%$. The optimized formulation increased the specific loaf volume from 1.7 to 2.8 cm^3^ g^−1^ and decreased crumb firmness from 10.6 to 3.7 N compared to the initial gluten-free bread formulation (C1). Egg white, milk powder, and psyllium contribute to the formation of a gluten-like network, which enables gas retention, dough expansion, and volume increase. In addition, soy lecithin, among hydrocolloids, enhances dough stability and moisture retention, resulting in a softer crumb. Sensory evaluation indicated good consumer acceptability (average score of 7 on a 9-point hedonic scale), particularly for texture and flavor. These findings suggest that optimal formulation of sorghum achieves both technological and sensory properties, supporting its potential as a viable gluten-free bread alternative.

## 1. Introduction

Sorghum is classified as a C4 plant, which enables it to grow in arid climates with high light intensity, tolerate water stress, and utilize water and CO_2_ efficiently [1]. Globally, approximately 46–50% of sorghum production is consumed as food. Sorghum is an excellent source of carbohydrates, proteins, phytochemical compounds (e.g., phenolic acids and flavonoids), vitamins—mainly B-complex such as thiamine, riboflavin, and niacin—and minerals including calcium, magnesium, iron, potassium, and zinc [2]. Whole sorghum retains the bran and germ, thereby providing dietary fiber, minerals, and phenolic compounds, which are lost during the refining process. In addition, whole sorghum exhibits technological properties such as water absorption and solubility—attributed to its fiber and protein content—and higher viscosity [3,4,5,6]. Sorghum’s phenolic profile provides multiple health benefits to consumers, including the prevention of diabetes and obesity, reduction in cholesterol levels, and the prevention of cardiovascular disease and certain types of cancer [7]. Additionally, sorghum flour is gluten-free, making it a suitable substitute for wheat flour in the diets of individuals with celiac disease.

Gluten is a complex mixture of proteins, primarily gliadin and glutenin, found in certain cereal grains such as wheat, triticale, barley, and rye. The gluten in wheat is essential for developing the rheological and structural properties of dough, including extensibility, resistance to stretching, mixing tolerance, and gas-holding capacity, all of which are crucial for bread-making [8,9].

The absence of gluten in sorghum flour presents technological challenges, leading to baked products that are rigid, exhibit irregular and brittle textures, and have low loaf volume. To address these limitations, the industry has employed various combinations of ingredients to enhance the rheological properties of dough and improve the quality of baked products. These ingredients include refined cereals (maize, rice, sorghum), pseudocereal flours (amaranth, buckwheat), and starches (potato, cassava), as well as hydrocolloids (xanthan gum and hydroxypropyl methylcellulose), emulsifiers (diacetyl tartaric ester of monoglycerides [DATEM], sodium stearyl-2-lactylate [SSL]), enzymes (amylases and proteases), and proteins (milk and egg) [10]. Schober et al. [11] evaluated the influence of xanthan gum and skimmed milk powder on the quality of gluten-free bread (GFB) formulated with a base of 70% sorghum flour and 30% cornstarch. They observed that inadequate levels of these ingredients can negatively affect the crumb structure, leading to reduced bread height, top collapse, increased baking loss, and decreased cohesion. The interaction of xanthan gum with additives such as SSL, DATEM, and flours, including rice and coconut flour, demonstrated positive effects on bread volume and crumb hardness [12,13]. Admassu Emire & Demelash Tiruneh [14] optimized and evaluated the incorporation of egg albumin and powdered milk into a base comprising 50% sorghum, 20% chickpea flour, 20% corn flour, and 10% potato flour. Their results indicated that the combination of egg albumin and milk powder enhanced both the sensory and technological qualities of GFB, producing a softer crumb characterized by small holes and thin cell walls, as well as increased volume. Onyango et al. [15] examined the addition of 6.7% *w*/*w* egg white powder to sorghum-based GFB, observing that egg whites mitigated textural defects associated with gluten absence—such as loose, disintegrating, coarse crumb, collapsed crust, flying top, and cracked crust—thereby facilitating an increase in specific volume up to 2.7 cm^3^g^−1^. Egg white proteins exhibit strong cohesive properties in addition to foaming capacity and stability, which may influence not only the rheological characteristics but also enhance both sensory and nutritional attributes [16].

On the other hand, lecithin functions as an emulsifier and surface-active agent that strengthens dough by interacting with gluten proteins, lipids, and starch. This interaction stabilizes gas cell walls and improves the dough’s gas retention capacity. Additionally, lecithin acts as an anti-firming agent by forming amylose–lipid complexes with gelatinized starch. These complexes inhibit starch retrogradation and delay the crystallization of amylopectin, a process attributed to the high lysophospholipid content of lecithin [17,18,19].

The use of psyllium husk powder has also been evaluated to improve the structural integrity of the crumb, appearance, texture, and shelf life of GFB, thereby increasing the specific loaf volume and decreasing the crumb firmness. It is due to the starch–psyllium matrix that enhances gas retention and strengthening of expanding cell boundaries. Additionally, psyllium acts as an effective moisture binder, helping to delay moisture loss [20]. Furthermore, psyllium’s water-holding capacity reduces starch digestion by limiting the mobility of enzymes within the digestive medium [21].

However, further research is needed to investigate the effects of multiple ingredients such as egg white, powdered milk, sugar, yeast, psyllium, xanthan gum, and soy lecithin on the quality attributes of GFB prepared with whole sorghum flour. Moreover, optimizing the levels of these ingredients is essential to maximize loaf volume and minimize crumb firmness. The mathematical and statistical approach known as response surface methodology (RSM) has been employed to optimize GFB formulations. This methodology is an effective tool for addressing multivariate problems and optimizing multiple responses within various experimental designs, as it can simultaneously evaluate multiple factors at different levels and their interactions while requiring a relatively small number of observations [22].

This study aimed to apply RSM to optimize whole sorghum flour-based formulations for producing GFB. Four consecutive optimization phases were conducted, each focusing on different groups of ingredients: protein-based components (egg white and powdered milk), hydrocolloid/fiber-based components (psyllium), and hydrocolloid/emulsifier-based components (xanthan gum and soy lecithin). The optimization targeted two key parameters: specific loaf volume and crumb firmness, which are recognized indicators of bread quality and consumer acceptability. This focus was chosen because GFB typically exhibits lower loaf volumes and crumb firmness values that are two to five times higher than those observed in wheat bread, significantly reducing consumer preference [8,23]. An additional aspect of this study is the utilization of whole sorghum flour instead of refined flour. Given that gluten-free products frequently incorporate ingredients that are low in protein and fiber but high in sugar and salt, the ingredients selected for this study were chosen not only for their potential to replace gluten and enhance technological functionality but also to improve the nutritional profile and sensory qualities of the final product.

## 2. Materials and Methods

### 2.1. Raw Materials

Sorghum (*Sorghum bicolor*) grains were purchased from “Semillas Las Huertas” (Mexico City, Mexico). The whole grains were cleaned manually to remove foreign material and damage. The cleaned grains were milled using a Homend 500 g multifunction grain mill (2500 W, 36,000 rpm, stainless steel, 70–300 mesh; Homend, Beijing, China) to obtain whole flour with a particle size of less than 250 µm. Whole sorghum flour was placed in polyethylene bags and stored at −20 °C until use. Maize starch (Maizena^®^, Unilever, Mexico City, Mexico), egg white (Huevo San Juan^®^, San Juan de los Lagos, Jalisco, Mexico), milk powder (Alpura^®^, Cuautitlán Izcalli, Mexico City, Mexico), yeast (*Saccharomyces cerevisiae*; Saf-Instant^®^, Lesaffre, Marcq-en-Baroeul, France), sugar (Zulka^®^, Culiacán, Sinaloa, Mexico), psyllium husk (*Plantago ovata*; Diquítra^®^, Tlalnepantla de Baz, Mexico City, Mexico), xanthan gum (Diquítra^®^, Tlalnepantla de Baz, Mexico City, Mexico), soy lecithin (Mi Granero^®^, San Pedro Cholula, Puebla, Mexico), salt (La Fina^®^, Mexico city, Mexico), and olive oil (Nutrioli^®^, Monterrey, Nuevo León, Mexico ). All the ingredients were obtained from Superfoods Mexico, a local supplier.

### 2.2. Proximate Compositions

The proximate compositions were determined for whole-sorghum flour in duplicate according to AACC-approved methods [24]: moisture (Method 44-19.01), ash (Method 08-03.01), crude protein (Method 46-13.01), crude fat (Method 30-25.01), and crude fiber (Method 32-10.01). Total carbohydrate content was calculated on a dry weight basis by difference.

### 2.3. Bread Making

The whole sorghum GFB was developed using the methodology proposed by Canelo-Álvarez et al. [25], with modifications based on preliminary trials. Preliminary experiments guided the selection of ingredients. The formulation was standardized based on 100 g of whole sorghum flour, to which the following components were added: 20 g of maize starch, 134 mL of egg white (EW), 10 g of milk powder (MP), 1.6 g of yeast, 8 g of sugar, 4 g of psyllium husk (PsH), 4 g of xanthan gum (XG), 4 g of soy lecithin (SL), 160 g of water, 2 g of salt, and 10 g of olive oil. This formulation was designated as the positive control (C1). The negative control (C2) was established by applying the AACC 10-10.03 method with modifications, in which wheat flour was replaced with whole sorghum flour, and the water content was adjusted to 80 mL per 100 g of flour. Furthermore, a wheat bread (C3) was prepared according to the AACC 10-10.03 method. All formulations were scaled to 50 g of flour per loaf to facilitate testing under conditions of limited material. All ingredients were mixed in a KitchenAid Professional 600 mixer (Whirlpool Co., Benton Harbor, MI, USA) at 125 RPM for 10 min. The mixture was then poured into rectangular baking pans (16.0 × 9.0 × 4.5 cm). Fermentation was carried out at 32 ± 2 °C for 60 min. Baking was performed in an air fryer oven (Gourmia model GTF7660, The Steelstone Group, Brooklyn, NY, USA) at 190 °C for 40 min. After baking, loaves were cooled on racks at room temperature to assess the physical quality of the whole sorghum GFB (Section 2.4). Finally, bread loaves were packaged in plastic bags and stored at room temperature until textural analysis was conducted 24 h after baking.

### 2.4. Experimental Design and Statistical Analysis

A central composite design (CCD) with an RSM was used to examine the effects and interactions of the ingredients to optimize the C1 formulation. The ingredients were classified into four optimization categories. Each variable to be optimized was coded with a five-level design. RSM was used to maximize the specific loaf volume ($Y_{1}$, cm^3^ g^−1^) and minimize the crumb firmness ($Y_{2}$, N) (Table 1). Both of the responses are primary quality indicators of GFB [23]. Five replicates were made at the center point, and three replicates were made at each of the points (−1.4, −1, 1, 1.4) of the design to estimate the sum of the squares of the error. The experimental data were analyzed using two software packages: Minitab statistical software (version 20, Minitab Inc., State College, PA, USA) and MATLAB (version R2015a, The MathWorks Inc., Natick, MA, USA) to plot the response surfaces. Starting with formulation C1, the different groups of ingredients were optimized sequentially to obtain formulations F1, F2, F3, and F4. The ingredient levels from the previous optimal formulation were maintained constant throughout the process.

A second-order polynomial regression analysis was applied to the experimental data obtained from RSM to estimate the coefficients of the linear and quadratic terms in the model for each response variable (Equation (1)). An analysis of variance (ANOVA) based on the CCD was conducted to determine the *p*-value, identifying which terms had a statistically significant effect on the responses. Additionally, Pareto charts were generated alongside ANOVA results to visualize and compare the significance of the effects on the response variables. The model’s fitting was evaluated based on the coefficient of determination $\left(R^{2}\right)$, the adjusted $R^{2}$ ($R_{adj}^{2}$), and the predicted $R^{2}$ ($R_{pred}^{2}$). $R^{2}$ indicates the proportion of variance in the variables that the model explains; $R_{adj}^{2}$ is calculated by considering the number of parameters, providing a more accurate measure when comparing models with different numbers of terms. $R_{pred}^{2}$ is used to estimate the proportion of variance in the response that the model would explain if it were applied to new observations or unused experimental data during the fitting process.

(1) $$y=\beta_{0}+\beta_{1}X_{1}+\beta_{2}X_{2}+\beta_{11}X_{1}^{2}+\beta_{22}X_{2}^{2}+\beta_{12}X_{12}+\varepsilon$$

where y is the response variable, $\beta_{0}$ is the intercept, which represents the fitted response at the center point of the design; $\beta_{1}$ and $\beta_{2}$ are the linear regression coefficients, $\beta_{11}$ and $\beta_{22}$ are the quadratic regression coefficients, and $\beta_{12}$ is the regression coefficient of the cross-product, that is, the interaction effect between the factors $X_{1}$ and $X_{2}$, and ε expresses the residual error that represents the unexplained variation in response.

### 2.5. Evaluation of Gluten-Free Bread Quality

The sorghum GFB volume (cm^3^) was determined using the seed displacement AACC 10-05.01 method. Specific loaf volume (cm^3^ g^−1^) was calculated as the ratio of bread volume to weight (g). The texture profile of the bread crumb was evaluated 24 h after baking using the AACC method 74-09.01, with a texture analyzer (Stable Micro Systems TA.XT Plus, Surrey, UK; software version 6.2) equipped with a 25 mm diameter cylindrical aluminum probe (P/25). Bread loaves were sliced into 12.5 mm thick sections, and two slices were stacked to achieve a uniform sample height. Samples were compressed to 40% deformation at a constant test speed of 1.7 mm/s, preceded by a pre-test speed of 1.0 mm/s and followed by a post-test speed of 10.0 mm/s. Six slices were evaluated for each bread sample (*n* = 3 per treatment).

#### 2.5.1. Quantitative Descriptive Analysis (QDA)

To assess the sensory properties of the optimal sorghum GFB formulation, a panel of seven trained evaluators rated the intensity of a whole bread slice using a scale from zero (“absent”) to five (“very intense”). The attributes evaluated included flavor (sweet, salty, acidic, and bitter), aroma (acetic, burnt, caramel, cereal, fermented, smoked, dairy, sourdough, toasted, honey, hazelnut/nutty, egg, fiber, and oily), texture (compact, crunchy, hard, and brittle crust; elastic, moist, soft, dry, and uniform crumb), and appearance (porosity, crumb, crust, and softness). Each panelist received one slice (12.5 mm thick) per session, served at room temperature (25 ± 2 °C) on a white ceramic plate. Samples were coded with three-digit random numbers and presented in a balanced order across panelists to minimize order effects. Evaluations were conducted in two separate sessions, with panelists unaware that both sessions involved the same product.

#### 2.5.2. Overall Acceptability

The consumer acceptance of the sorghum GFB, prepared using the optimal formulation, was evaluated using a 9-point hedonic scale (1 = extremely unpleasant to 9 = extremely pleasant). One hundred and ten consumers (40 men and 70 women) aged 18 to 30 years voluntarily participated in the study. Participants were first screened to confirm the absence of allergies or intolerances to any ingredients in the product. Each consumer received a quarter slice of bread, including both crumb and crust, served at room temperature on a disposable plate. Samples were coded with random three-digit numbers. The bread sample was presented individually to the panelists, who were then asked to rate their overall liking using a hedonic scale.

### 2.6. Statistical Analysis

The data were analyzed using statistical analysis of variance (ANOVA) with JMP software (version 16, SAS Institute, Cary, NC, USA). The bread quality parameters were assessed in triplicate, and the results were expressed as mean values ± standard deviation. Tukey tests were used to evaluate significant differences between means (p<0.05). 

## 3. Results and Discussion

### 3.1. Proximal Composition of Whole Sorghum-Flour

The chemical composition of whole sorghum flour is presented in Table 2. Like other cereal grains, the main component of sorghum is carbohydrates in the form of starch (85.37% d.b.). The role of starch during baking is to bind the water to create a gas-permeable structure and gelatinization. Higher starch gelatinization temperatures lead to higher final bread volume, changing a fluid and aerated batter to a solid and porous structure. Also, starch presents a high influence on the dough parameters, texture, moisture retention, and final quality [26,27,28]. The protein content (8.35% d.b.) is lower than that reported for whole wheat flour (14.01%) and the average reported for sorghum by other authors (11%) [7,29]. This variability may be attributed to different factors, such as the sorghum variety and its cultivation. Sorghum storage proteins, called kafirins, are a type of hydrophobic protein encapsulated within protein bodies that remain intact during cooking at low shear rates, rendering them generally less digestible and unavailable to form functional structures in foods. Additionally, kafirins are excessively short and strangely reticulated, which hinders protein–protein interactions and impairs the cohesiveness and gas-holding capacity of sorghum doughs [30,31]. These structural and functional limitations reduce the technological performance of sorghum in bakery applications, particularly affecting dough viscoelasticity, crumb structure, and overall product quality. To overcome these challenges, incorporating complementary protein sources, such as milk powder and egg white, may establish intermolecular interactions through various mechanisms—including hydrogen bonding, electrostatic interactions, and protein-protein cross-linking—with sorghum proteins, thereby enhancing dough rheology and final product quality. The crude fat content (3.5% d.b.) was similar to that reported by Nieto-Mazzocco et al. [29] for sorghum flour and higher than that of whole wheat flour. Lipid fractions contribute to the stability of bubbles generated during baking before starch gelatinization. This stabilization enhances dough structure, promotes a finer crumb texture, and influences key sensory attributes of bakery products [32]. The fiber content (1.4% d.b.) falls within the range reported by some authors [33,34]; however, it is relatively low compared to other cereals, such as wheat. This limitation negatively affects optimal dough functionality and bread quality. To address this issue, incorporating hydrocolloids, such as psyllium, is essential to compensate for the lack of gluten, interact with system proteins, and improve loaf volume, texture, and overall sensory acceptability of gluten-free breads.

### 3.2. Evaluation of the Fitted Model

**Figure 1:**
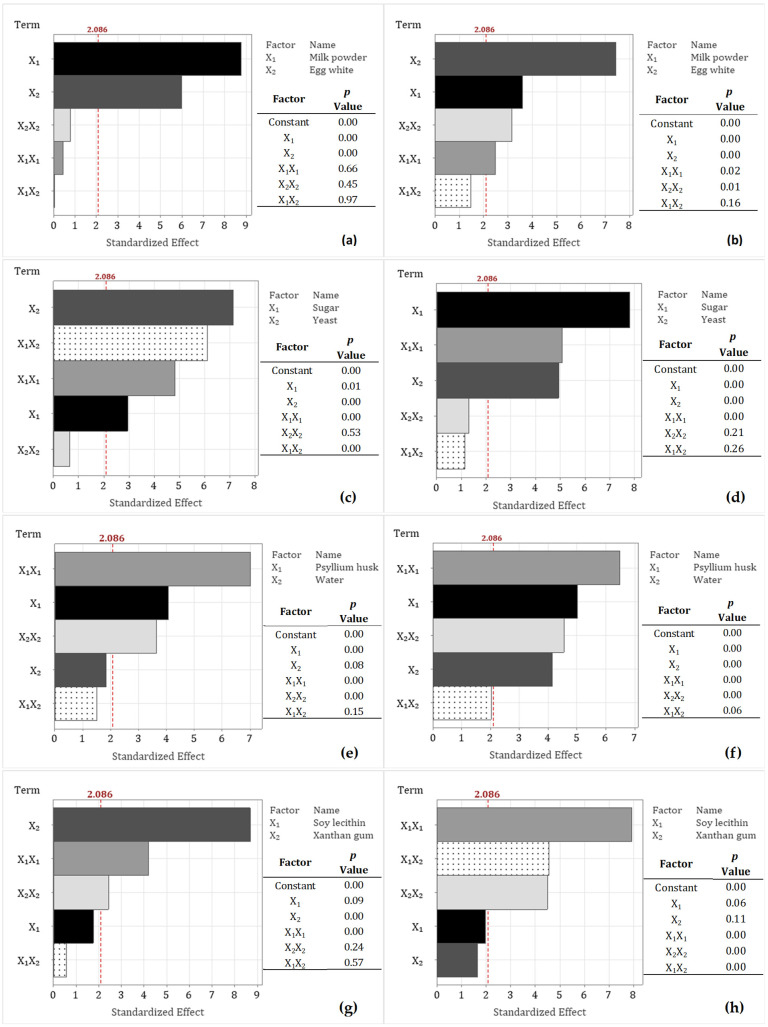
Pareto charts of the standardized effects of ingredients on specific volume (cm^3^ g^−1^, left) and crumb firmness (N, right), along with the *p*-values of terms in the generalized quadratic model: (**a**,**b**) milk powder–egg white, (**c**,**d**) yeast–sugar, (**e**,**f**) psyllium husk–water, (**g**,**h**) soy lecithin–xanthan gum Factors (horizontal bars) that cross the reference line (2.086) are statistically significant (p< 0.05).

Using the CCD, multiple regression analysis, and ANOVA, experimental data for each treatment were analyzed. The *p*-value indicated which parameters had a significant effect on the adjusted model (Figure 1). A *p*-value of less than 0.05 (*p* < 0.05) suggests that the parameter has significant differences. In all the fitted models, the constant (intercept) term was found to be statistically significant. $R^{2}$ values were greater than 80% for all models (Table 3). The closer the $R^{2}$ value is to unity, the more accurate the response, adequate fit, and repeatability that could be predicted [35,36]. Nevertheless, according to Myers et al. [36], $R^{2}$ values greater than 75% are also significant and suggest that the model is well-adjusted. The $R_{adj}^{2}$ does not differ more than 6% from the calculated $R^{2}$ in all cases, which indicates a good adaptation of the theoretical values for the experimental data of the model, and the possibility of non-significant terms being included in the model is low [37,38,39,40]. The mean of $R_{pred}^{2}$ was 70.8%. A low $R_{pred}^{2}$ value and a considerable difference from $R_{adj}^{2}$ may indicate a decrease in the predictive capacity of the developed model concerning a new set of data despite maintaining a good intercorrelation. This effect may be associated with all the variables involved in the development of bread, such as atmospheric humidity, number of viable yeast cells, and heat transfer during baking, among others. However, according to Veerasamy et al. [40], a value or $R_{pred}^{2}$ greater than 60% is an indicator of good external predictability.

### 3.3. Effect of the Ingredients on Whole Sorghum on Bread Making Quality

#### 3.3.1. Milk Powder (MP) and Egg White (EW)

**Figure 2:**
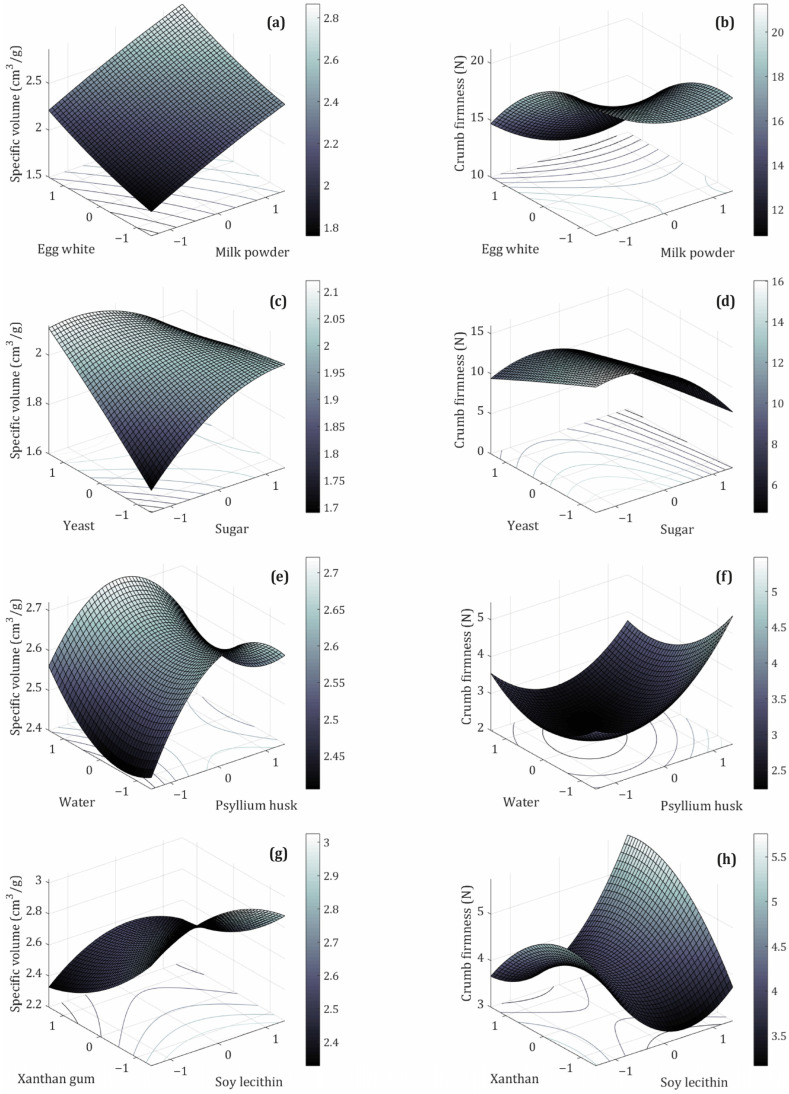
Effects of the different ingredients’ optimization categories: (**a,b**) milk powder–egg white, (**c,d**) yeast–sugar, (**e,f**) psyllium husk–water, (**g,h**) soy lecithin–xanthan gum, on specific volume and crumb firmness of whole sorghum GFB, respectively.

MP and EW are high-quality animal protein sources widely used in GFB formulations to enhance the amino acid profile and improve overall nutritional value. MP contains approximately 25–27% protein, with casein as the predominant protein fraction. EW contains about 10% protein (m.b.), with ovalbumin comprising approximately 54% of its total protein content [41,42,43]. The coded levels of MP and EW evaluated, along with the responses—specific loaf volume and crumb firmness—are shown in Figure 2a and Figure 2b, respectively. An increasing trend in specific volume was observed with higher contents of both ingredients (Figure 2a). Simultaneously, crumb firmness exhibited a decreasing trend, mainly associated with higher concentrations of EW, particularly at levels of 1.0 and 1.4 (Figure 2b). This behavior is attributed to the proteins in MP and EW, which have good solubility as well as high emulsifying and foaming capacities, allowing the formation of protein networks and a viscoelastic structure similar to gluten, thereby improving loaf volume [41]. Furthermore, EW proteins are globular with highly hydrophilic surfaces that effectively bind water, enabling the formation of strong, cohesive, viscoelastic films essential for foam stability and improved gas retention in GFB. EW also contains cysteine, which, like wheat proteins, can form temperature-stable cross-links and stable edible gels upon thermal activation [42,43,44]. Similar effects have been reported by other authors, such as Admassu Emire & Demelash Tiruneh [14], who observed that adding egg albumin and powdered milk to a base of sorghum (50%), chickpea flour (20%), corn flour (20%), and potato flour (10%) positively affects the sensory and technological quality of GFB, resulting in a softer crumb with small air cells and thin cell walls, while also improving volume.

The Pareto charts showed the effects of MP and EW on specific volume and crumb firmness are presented in Figure 1a and Figure 1b, respectively. These charts help identify the most significant factors and interaction effects for process optimization. Absolute values exceeding the reference line of 2.086 are considered statistically significant (*p* ≤ 0.05) [45]. As shown in Figure 1a, only the linear terms had a positive effect on specific volume.

Regarding crumb firmness (Figure 1b), only the interaction terms between ingredients did not have a significant effect. This outcome may be attributed to the distinct mechanisms by which EW and MP influence dough structure. Casein proteins in MP exist as large micellar aggregates that contribute to moisture retention and promote a crumbly texture. In contrast, EW acts as a foam stabilizer and binder, enhancing the structure during baking. Additionally, in the GFB formulation, both ingredients, along with sorghum starch, compete for the limited water available during baking, which affects starch gelatinization and, consequently, crumb firmness [41,46].

#### 3.3.2. Yeast and Sugar

Figure 2c,d illustrate the relationship between the coded levels of yeast and sugar and the response variable. As expected, an increase in specific volume was observed, attributable to the role of yeast as a leavening agent that produces carbon dioxide (CO_2_), acetic acid, and ethanol. These fermentation products cause the dough to expand, thereby achieving the desired volume in the final product [47]. However, a reduction in specific volume was observed for certain combinations of coded yeast and sugar levels (0–1.4, 1–−1, 1–1, and 1.4–0, respectively). This decrease may result from elevated yeast concentrations (1, 1.4) or a central yeast level (0) combined with high sugar levels (1.4), which can lead to excessive CO_2_ production within a short timeframe. This over-proofing generates large bubbles that rise rapidly and subsequently collapse, failing to maintain a stable dough structure. In cases where sugar is not fully utilized as a substrate by yeast (0–1.4, 1–1), it can still influence the final product through alternative mechanisms, as observed by Chevallier et al. [48], who demonstrated, through macro- and microscopic analyses of dough and wheat biscuits, that sugar contributes to structural formation during baking by melting and forming bridges between protein aggregates and lipid components. This sugar-induced matrix enhances the mechanical strength and cohesiveness of the final product. Nonetheless, high sugar concentrations may compete with starch for limited water availability, potentially inhibiting starch gelatinization and thereby affecting crumb firmness and overall textural quality.

Pareto diagrams showed that the levels of yeast and sugar utilized in this study significantly affect specific volume (Figure 1c) and crumb firmness (Figure 1d). Although sugar primarily acts as a fermentable substrate for yeast, in the present work, sugar does not exhibit a relationship with yeast, indicating that these two components operate through independent mechanisms. Marti et al. [49] reported that the specific volume of GFB remains relatively constant across different sugar concentrations, suggesting minimal interaction effects. Moreover, it is proposed that the nonsignificant interaction terms between yeast and sugar may be explained by the range of sugar concentrations examined, which do not influence leavening capacity or CO_2_ production; this independence at elevated sugar levels has been documented by other studies [48,50]. Additionally, sugar may serve as an additive affecting dough hydration and starch gelatinization, which could contribute to the observed reduction in crumb firmness.

#### 3.3.3. Psyllium Husk (PsH) and Water

Figure 2e shows the effect of coded experimental conditions involving psyllium husk (PsH) and water on specific volume. The specific volume response exhibits a saddle point, with an initial increase followed by a decrease beyond a certain threshold. This saddle point resembles the lowest point on the central ridge of a horse saddle connecting two peaks [51]. The observed increase in specific volume is attributed to the functional properties of psyllium husk, which is a dietary fiber source for GFB. PsH acts as a hydrocolloid, improving dough quality through its gas- and water-holding, gelling, and structure-building properties [52]. Moreover, PsH can interact with system proteins via ionic and non-ionic interactions, affecting dough strength [53]. However, a decrease in specific volume was observed when high PsH levels (1.0 and 1.4) were combined with low water levels (−1 and 0). Previous studies have reported a reduction in loaf volume at higher PsH concentrations due to excess fiber interfering with gas retention and promoting the formation of a more rigid and less extensible matrix, thereby limiting dough expansion [54,55,56]. Gao et al. [57] reported similar behavior in a gluten-free buckwheat formulation, where specific volume initially increased due to enhanced gas-holding capacity but decreased beyond a 15% PsH proportion. This decrease in specific volume was attributed to increased water content in the dough. Water acts as a plasticizer within dough systems; however, at higher levels, it can weaken the matrix by diluting interactions among flour components, such as proteins and hydrocolloid networks. This weakening reduces gas retention capacity, leading to unstable bubbles and their subsequent coalescence during fermentation and baking. As a result, the dough exhibits a more porous but less cohesive structure, ultimately resulting in reduced loaf volume [58,59,60].

The Pareto chart (Figure 1e) indicates that the interaction between PsH and water does not significantly affect the specific volume of sorghum GFB. This outcome may be explained by competition for water among PsH and other constituents, such as sugar, starch, and sorghum fiber, which potentially interferes with PsH’s binding capacity, thereby reducing its ability to hydrate and form a gel. As a result, within the evaluated range of water absorption (124–154 mL/100 g sorghum flour), water content, considered as an independent variable, did not have a significant impact on the sorghum GFB samples. The importance of maintaining appropriate PsH and water levels is also observed in Figure 2f, where crumb firmness reaches a minimum (“valley point”) at a specific PsH value. When combined with optimal water levels, this PsH facilitates the formation of moisture-binding structures that reduce crumb firmness and enhance crumb resilience. This pattern, also observed in the specific volume, supports the hypothesis that these two factors are interrelated through PsH functionality and dough hydration [20,53]. Furthermore, the Pareto diagram (Figure 1f) indicates that both the linear and quadratic terms significantly affect crumb firmness. However, no significant interaction effect between these terms was observed. This non-interaction aligns with the results for specific volume, suggesting that while PsH and water levels independently affect both responses, their combined interaction does not have a significant effect within the parameter range investigated in this study.

#### 3.3.4. Soy Lecithin (SL) and Xanthan Gum (XG)

The effects of SL and XG on the specific volume of sorghum GFB are illustrated in Figure 2g. An increase in XG concentration is associated with a decrease in specific volume. The Pareto chart (Figure 1g) demonstrates that XG concentration significantly influences the specific volume of sorghum GFB, with the linear term showing the most pronounced effect. As a hydrocolloid, XG behaves similarly to PsH, contributing to improved dough rheology by enhancing water retention, viscosity, and gas-holding capacity. In contrast, although SL functions as an emulsifier within GFB matrices—interacting with fat, starch, protein, and water to form complexes that strengthen the dough and promote CO_2_ retention [56,61]—it did not exhibit a statistically significant effect on specific volume in the present study. This lack of effect can be attributed to the predominant influence of hydrocolloid ingredients (PsH and XG) on the sorghum GFB structure, which interact with other components to facilitate the formation of gluten-like networks under the conditions examined.

The observed decrease in specific volume of sorghum-based gluten-free bread (GFB) with increasing concentrations of xanthan gum (XG) can be attributed to several factors. These include an increase in viscosity, which may hinder gas retention during fermentation; enhanced dough strength; and an imbalance between the available water and xanthan gum, resulting in a denser dough that may not sufficiently expand during baking [62,63]. Similar behavior was reported by Madhuresh et al. [63], who observed that the interaction between XG and other ingredients, such as cornstarch, decreases the gas retention capacity of gluten-free batters and modifies the rheological properties, leading to reduced consistency and increased plasticity.

Furthermore, an increase in the level of XG leads to a decrease in crumb firmness (Figure 2h). Simultaneously, the response to the concentration of SL exhibits a “bending” behavior. At a specific concentration (−1, 0), SL is capable of forming a lipid–starch or lipid–protein complex that contributes to bread softening; however, levels at −1, −1.4, and 1.4 are associated with increased crumb firmness. Abdel-Gawad et al. [62] similarly reported an increase in crumb firmness with higher gum concentrations, which produce more cohesive doughs that enhance bread firmness. In contrast, Ren et al. [64] found that the emulsifying properties of SL improve gas retention during baking and promote a more uniform distribution of fat and moisture within the dough, thereby contributing to a firmer and more homogeneous crumb structure.

The Pareto chart for crumb firmness (Figure 1h) showed that neither SL nor XG significantly affects this parameter when analyzed as individual linear terms. Although water is often evaluated alongside XG and SL to optimize dough hydration and structure, the present study assessed water in combination with PsH to minimize the use of gums and additives and to emphasize ingredients rich in dietary fiber, thereby improving the nutritional quality of sorghum gluten-free bread (GFB). However, since both ingredients exhibit strong moisture retention properties, their influence on specific volume and crumb firmness may have been diminished, as the amount of available water was predetermined in the formulation.

### 3.4. Optimization and Analysis of the Optimized Formulations

The optimal ingredient levels, determined using a composite desirability score of 1.00, along with the corresponding specific volume and crumb firmness values, are presented in Table 4. The optimization process aimed to maximize specific volume and minimize crumb firmness, as these parameters are critical indicators of bread quality and consumer acceptability in gluten-free pan bread formulations. Compared with the control formulations, optimization with F1 and F2 resulted in a significant increase in specific volume—from 1.7 ± 0.4 (C1) and 0.9 ± 0.5 (C2) to 2.2 ± 0.1 cm^3^ g^−1^—and a significant reduction in crumb firmness—from 10.6 ± 0.0 (C1) and 34.2 ± 2.1 (C2) to 8.1 ± 0.4 and 7.6 ± 0.0 N, respectively. No significant differences were observed between the two optimized formulations. These changes may be attributed to the complementary roles of these ingredients. While yeast and sugar directly influence CO_2_ production during fermentation, MP and EW form networks that enhance dough structure and facilitate gas retention, which is crucial in GFB. Under the optimal conditions predicted by the F3 and F4 formulations, a significant increase in specific volume to 2.5 and 2.8 cm^3^ g^−1^ and a significant decrease in crumb firmness to 2.8 and 3.7 N were achieved, respectively. PsH and XG modify the viscoelastic properties by enhancing dough elasticity, gas retention, and dough stability. SL ensures uniform gas distribution, contributing to improved dough consistency. In combination, these ingredients form a strong and elastic network, resulting in a substantial increase in specific volume. The significant decrease in specific volume observed between the F3 and F4 optimizations can be explained by the PsH evaluation, which was carried out in the presence of fixed concentrations of SL and XG as defined in formulation C1. These levels exceeded the optimal values identified in the present study. As demonstrated in the F4 optimization, excessive concentrations of these hydrocolloids and emulsifiers increase viscosity and reduce dough expansion during baking, ultimately limiting loaf volume. In the optimized formulation F4, no significant difference in crumb firmness was observed compared to the wheat-based control (C3). This finding suggests that the technological functionality achieved within the gluten-free matrix was sufficient to produce a crumb texture comparable to that of traditional wheat bread. However, the loaf volume of F4 remained significantly lower than that of C3, highlighting the inherent structural limitations of gluten-free systems, where the absence of a viscoelastic gluten network restricts gas retention and expansion during proofing and baking.

### 3.5. Sensory Evaluation

**Figure 3:**
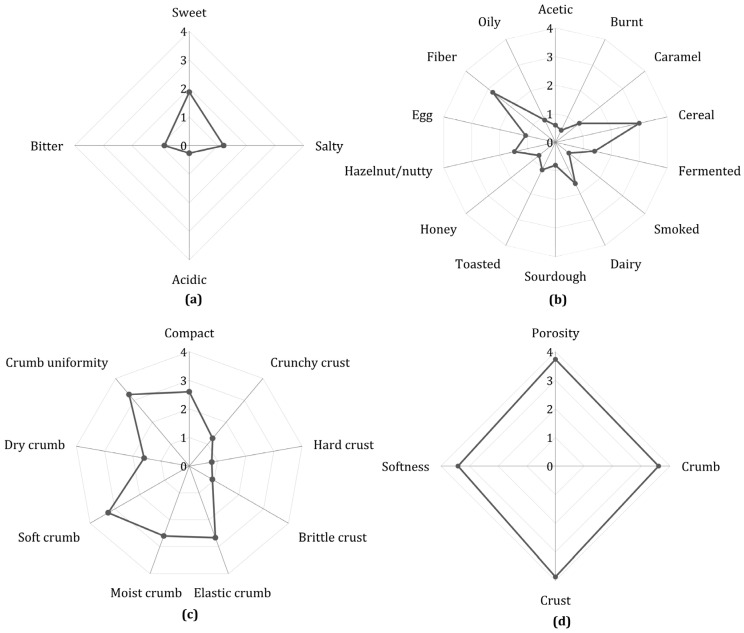
Sensory evaluation of optimized GFB. (**a**) Flavor; (**b**) aroma; (**c**) texture; (**d**) appearance.

Sensory evaluation was conducted on the F4 formulation, as this sample contained all the optimized ingredients. Panelists detected aromas such as caramel (1.1) and honey (0.7), indicating a low degree of sugar caramelization. This finding is consistent with the low intensities recorded for toasted (1.1) and burnt (0.4) attributes (Figure 3b). Similarly, in the profile of the four basic tastes (Figure 3a), sweetness (1.9) was perceived more strongly than saltiness (1.2), confirming a mild and balanced flavor profile. The sample also scored 2.8 for fiber and cereal flavor, which aligns with consumer observations that the bread’s taste resembled that of whole wheat bread. Within this profile, the protein-based ingredients contributed low intensities for dairy and butter (1.6) and egg (1.1) notes (Figure 3b), suggesting that their incorporation did not result in overpowering off-flavors. Although consumers found the flavor pleasant, 10% reported that the bread tasted slightly bland. These characteristics of GFB have been reported by other authors [65,66], who also observed that while GFB can achieve good consumer acceptance, it is often perceived as having a milder flavor compared to traditional wheat-based options. Additionally, consumers described the bread as spongy, soft, and moist, highlighting its desirable texture. This aligns with the judges’ evaluations (Figure 3c), who reported a uniform crumb (3.3) and compactness (2.6), as well as moistness (2.6), elasticity (2.7), and softness (3.3). The appearance (Figure 3d), including crumb, crust, softness, and porosity, was rated close to a score of 4.0.

**Figure 4:**
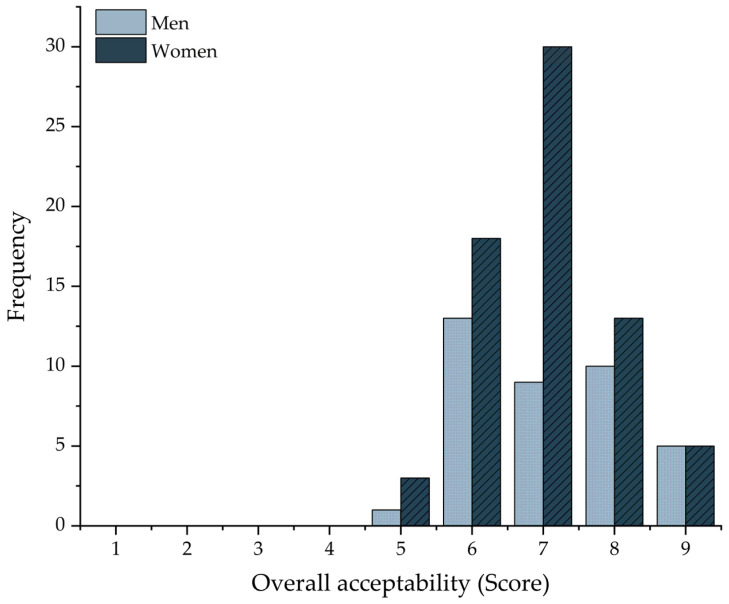
Overall acceptability scores (9-point hedonic scale) of the optimized sorghum GFB (F4), presented by gender. Data are expressed as mean values (*n* = 110).

The optimal sorghum GFB formulation (F4) achieved an average score of 7.04 ± 0.1 out of 9 on the hedonic scale, with a higher frequency of responses clustered at score 7 (Figure 4). When analyzed by gender, male participants reported an average score of 7.13 ± 1.1, while female participants rated the bread at 6.9 ± 1.0. All participants were regular consumers of wheat bread and reported no allergies or dietary restrictions, indicating a satisfactory level of consumer acceptance. Furthermore, sensory evaluation confirmed the product’s acceptability, suggesting its potential as a viable option for gluten-intolerant individuals and those seeking breads rich in protein and fiber.

## 4. Conclusions

This study optimized a whole sorghum GFB formulation using response surface methodology. The interaction effects of the ingredients—milk powder, egg white, yeast, sugar, psyllium, water, soy lecithin, and xanthan gum—significantly improved the overall bread quality. After four consecutive optimizations, the optimized ingredient combination increased bread volume by 60% compared to formulation C1 (unoptimized) and by 200% compared to formulation C2 (without additives). Crumb firmness decreased by 65% and 95% compared to C1 and C2, respectively. These results indicated that the combination of proteins (mainly egg white) and hydrocolloids (psyllium and xanthan gum) contributed to the formation of a viscoelastic network. Additionally, the psyllium used limited the effect of xanthan gum, probably due to competition for water. Furthermore, sensory evaluation confirmed good acceptability. It suggests that sorghum is a viable alternative for the commercial production of gluten-free bread. The implemented methodology enables the continued evaluation of various combinations of ingredients and additives to improve the technological and sensory qualities of GFB, as well as its nutritional composition and other aspects of technological and industrial relevance.

## Figures and Tables

**Table 1:** Coded and actual levels of the factors in the CCD of the RSM *.

Formulations	Ingredients (g)	Term	Coded Levels				
			−1.4	−1	0	1	1.4
F1	MP	$X_{1}$	2.2	4.6	10.2	16.0	18.4
	EW (mL)	$X_{2}$	54.4	66.0	94.2	122.4	134.0
F2	Sugar	$X_{1}$	2.6	4.0	7.4	10.6	12.0
	Yeast	$X_{2}$	0.4	2.0	6.0	10.0	11.6
F3	PsH	$X_{1}$	4.0	5.0	7.6	10.0	11.0
	Water	$X_{2}$	125.8	130.0	140.0	150.0	154.2
F4	SL	$X_{1}$	0.4	1.2	3.0	4.8	5.6
	XG	$X_{2}$	0.4	1.2	3.0	4.8	5.6

* CCD: Central composite design; RSM: response surface methodology; MP: milk powder; EW: egg white; PsH: psyllium husk; SL: Soy lecithin; XG: xanthan gum. The quantities of the ingredients are specified per 100 g of whole-sorghum flour.

**Table 2:** Chemical composition of whole-sorghum flour.

Parameter	(%)
Moisture	9.70 ± 0.06
Crude Protein *	8.35 ± 0.02
Crude Fat *	3.5 ± 0.01
Crude Fiber *	1.4 ± 0.0
Ash *	1.3 ± 0.04
Carbohydrates *	85.37 ± 0.0

* Reported as dry based. Mean experimental values (±standard deviation, *n* = 2).

**Table 3:** Predicted model equations and determination coefficients *.

Formulation	Predicted Model Equations	$R^{2}$ (%)	$R_{adj}^{2}$ (%)	$R_{pred}^{2}$ (%)
Specific volume				
F1	${\hat{Y}}_{1}=2.30+0.23x_{1}+0.16x_{2}-0.01x_{1}x_{1}+0.02x_{2}x_{2}+0.00x_{1}x_{2}$	84.96	81.20	72.22
F2	${\hat{Y}}_{1}=2.02+0.02x_{1}+0.06x_{2}-0.04x_{1}x_{1}-0.01x_{2}x_{2}-0.07x_{1}x_{2}$	85.69	82.11	73.44
F3	${\hat{Y}}_{1}=2.62+0.04x_{1}+0.02x_{2}-0.07x_{1}x_{1}+0.04x_{2}x_{2}-0.02x_{1}x_{2}$	82.21	77.76	66.20
F4	${\hat{Y}}_{1}=2.70+0.03x_{1}-0.16x_{2}-0.08x_{1}x_{1}+0.05x_{2}x_{2}+0.02x_{1}x_{2}$	84.08	80.09	66.49
Crumb firmness				
F1	${\hat{Y}}_{2}=16.76-1.15x_{1}-2.39x_{2}+0.86x_{1}x_{1}-1.09x_{2}x_{2}-0.67x_{1}x_{2}$	81.65	77.06	69.25
F2	${\hat{Y}}_{2}=12.53-2.42x_{1}-1.53x_{2}-1.68x_{1}x_{1}-0.43x_{2}x_{2}+0.50x_{1}x_{2}$	84.94	81.18	70.52
F3	${\hat{Y}}_{2}=2.34+0.38x_{1}-0.31x_{2}+0.52x_{1}x_{1}+0.37x_{2}x_{2}-0.22x_{1}x_{2}$	83.62	79.53	67.42
F4	${\hat{Y}}_{2}=4.05+0.13x_{1}+0.11x_{2}+0.57x_{1}x_{1}-0.32x_{2}x_{2}+0.43x_{1}x_{2}$	85.80	82.25	76.07

* $R^{2}$: Coefficient of determination; $R_{adj}^{2}$: adjusted coefficient of determination; $R_{pred}^{2}$: predictive coefficient of determination. F1: milk powder ($X_{1}$)-egg white ($X_{2}$); F2: sugar ($X_{1}$)-yeast ($X_{2}$); F3: psyllium husk ($X_{1}$)-water ($X_{2}$); F4: soy lecithin ($X_{1}$)-xanthan gum ($X_{2}$).

**Table 4:** Optimal conditions and experimental data for the responses.

Optimization	Optimal Conditions		Specific Volume	Firmness Crumb
	$X_{1}$	$X_{2}$	(cm^3^ g^−1^)	(N)
C1	-	-	1.7 ± 0.4 e	10.6 ± 0.0 b
C2	-	-	0.9 ± 0.5 f	34.2 ± 2.1 a
C3	-	-	3.5 ± 0.0 a	5.8 ± 0.5 cd
F1	8.6	67	2.2 ± 0.1 d	8.1 ± 0.4 bc
F2	5.8	1.3	2.2 ± 0.3 d	7.6 ± 0.0 bc
F3	3.7	75.4	2.6 ± 0.0 c	2.7 ± 0.2 d
F4	1.9	0.2	2.8 ± 0.1 b	3.7 ± 0.3 d

Mean experimental values (± standard deviation, *n* = 3). C1: Control formulation without optimization; C2: sorghum AACC 10-10.03 formulation; C3: wheat AACC 10-10.03 formulation; F1: milk powder ($X_{1}$)-egg white ($X_{2}$); F2: sugar ($X_{1}$)-yeast ($X_{2}$); F3: psyllium husk ($X_{1}$)-water ($X_{2}$); F4: soy lecithin ($X_{1}$)-xanthan gum ($X_{2}$). Different letters within a column indicate significant differences (p < 0.05).

## Data Availability

The original contributions presented in this study are included in the article. Further inquiries can be directed to the corresponding authors.

## Abbreviations

The following abbreviations are used in this manuscript:

DATEM	Diacetyl tartaric ester of monoglycerides
SSL	Sodium stearyl-2-lactylate
GFB	Gluten-free bread
RSM	Response surface methodology
EW	Egg white
MP	Milk powder
XG	Xanthan gum
SL	Soy lecithin
W	Water
S	Sugar
Y	Yeast
CCD	Central composite design
